# The Role of Screening for Venous Thromboembolism in Pelvic Trauma Patients: A Single-Centre Retrospective Study

**DOI:** 10.3390/jcm13216347

**Published:** 2024-10-23

**Authors:** Grzegorz Doroszewski, Marcin Kurzyna, Adam Caban

**Affiliations:** 1 Pelvic Injury and Pathology Department, Centre of Postgraduate Medical Education, Gruca Orthopedic and Trauma Teaching Hospital, Konarskiego 13, 05-400 Otwock, Poland; 2 Chair and Department of Pulmonary Circulation, Thromboembolic Diseases and Cardiology, Center of Postgraduate Medical Education, European Health Center, Borowa 14/18, 05-400 Otwock, Poland

**Keywords:** venous thromboembolism, deep vein thrombosis, pulmonary embolism, screening, pelvis, acetabulum, fractures

## Abstract

**Background:** Venous thromboembolism (VTE) is a severe complication following pelvic trauma. Thromboprophylaxis has reduced the risk of VTE in the pelvic trauma population; nevertheless, the risk remains high. A substantial pulmonary embolism has emerged as the unexpected cause of death among patients presenting with ‘minor pelvic fractures’. The purpose of this study was to analyse the single-centre experience with a surveillance protocol. We hypothesised that DVT surveillance with VUS and CTPA may reduce the perioperative mortality–morbidity rate in the subpopulation of patients with pelvic or acetabular fractures. **Methods:** This is a retrospective analysis of patients admitted with pelvic or acetabular fractures between January 2001 and December 2021. In April 2013, a screening protocol for VTE was introduced. This protocol included venous ultrasound and CTPA for patients with chest injuries. Patients from two groups—one screened for VTE and one without screening—were compared. **Results:** Of the 3186 patients with pelvic and/or acetabular fractures who were admitted, 1975 were not screened for VTE and 1211 underwent screening. There were more VTE cases in the screening group (5.62% vs. 0.86%, *p* < 0.001). Nine (0.46%) cases of sudden death occurred in the non-screening group, and all of them died with symptoms of acute PE. Since the screening was introduced, we have not encountered any deaths in the perioperative period (0.46 vs. 0, *p* = 0.02). **Conclusions:** The combined use of VUS and CTPA in chest-injured patients is a trustworthy means of screening for VTE, resulting in decreased mortality rates for those with pelvic and acetabular fractures by accurately diagnosing VTE during the perioperative phase.

## 1. Introduction

Most cases of pelvic ring and acetabular fractures are the result of high-energy blunt trauma [1]. Venous thromboembolism (VTE) is a severe complication following pelvic trauma.

Without chemoprophylaxis, up to 61% of patients with pelvic fractures are diagnosed with deep vein thrombosis (DVT) [2,3]. Pulmonary embolism (PE) is the most common cause of death in trauma patients who survive more than seven days [4]. Moreover, even patients with ‘minor fractures of the pelvis’ die unexpectedly due to a massive pulmonary embolus [5]. Thromboprophylaxis has reduced the risk of VTE in the pelvic trauma population; nevertheless, the risk remains high, varying from 5% to 38% [6,7].

Currently, there are different approaches regarding thromboprophylaxis, screening tests after pelvic trauma and treatment protocols for patients diagnosed with VTE worldwide [8,9,10,11,12,13]. Venography was once the method of choice for a diagnosis of DVT [2,14], but owing to its invasiveness and use of potentially hazardous contrast agents, it was gradually replaced by non-invasive methods such as ultrasound or magnetic resonance [14].

In our department, a routine screening protocol with venous ultrasound (VUS) for all patients with pelvic or acetabular fractures and a computed tomography pulmonary angiogram (CTPA) for patients with additional thoracic injuries or clinical symptoms of PE was introduced in 2013. The purpose of this study was to analyse our single-centre experience with this surveillance protocol. We hypothesised that DVT surveillance with VUS and CTPA may reduce the perioperative mortality–morbidity rate in the subpopulation of patients with pelvic or acetabular fractures.

## 2. Materials and Methods

### 2.1. Department Data

This study was conducted in the Department of Pelvic Trauma and Pathology at the tertiary health care Centre of Postgraduate Medical Education in Otwock, Warszawa (Poland). The department acts as a regional referral centre for the care of patients with pelvic and acetabular fractures.

We performed a retrospective study to evaluate a routine screening VUS protocol in patients with pelvic fractures. Both the paper and electronic medical records of patients with pelvic and acetabular fractures who were admitted to the Department of Pelvis Injury and Pathologies of the Centre of Postgraduate Medical Education between January 2001 and December 2021 were reviewed. The inclusion criteria were as follows: (1) patients with pelvic or acetabular fractures; (2) age older than 18 years. Patients with a history of VTE or diagnosed and treated at the time of admission were excluded.

The patients were divided into two groups: the screening group included patients admitted from April 2013 to December 2021 who were screened for VTE, and the non-screening group comprised patients admitted from January 2001 to March 2013 (control group) without a screening protocol for VTE.

### 2.2. Anticoagulant Prevention of VTE

In both groups, all patients received low-molecular-weight heparin (LMWH) primary prophylaxis upon admission to the department or continued it if they were transferred from other hospitals unless contraindicated (enoxaparin (Sanofi Winthrop Industrie, Gentilly, France), which was administered as 40 mg by subcutaneous injection, once daily; deltaparin (Pfizer Europe, Bruxelles, Belgium), 5000 IU anti-Xa/0.2 mL by subcutaneous injection, once daily; nadroparine, 0.2 mL (GlaxoSmithKline Export Middlesex, Great Britain) (1900 IU anti-Xa) in patients weighing <51 kg or 0.3 mL (2850 IU anti-Xa) in patients weighing <70 kg or 0.4 mL (3800 IU anti-Xa) in patients weighing ≥70 kg by subcutaneous injection, once daily). The anticoagulant prevention of VTE was conducted for 35 days or until the patient was mobilised. In cases where DVT and/or PE were diagnosed, therapeutic doses of enoxaparin were used (1 mg/kg every 12 h or 1.5 mg/kg daily by subcutaneous injection) according to Polish guidelines [15].

### 2.3. Screening Protocol

In the screening group, all patients underwent examinations for VTE. The screening protocol included a duplex ultrasound examination of pelvic veins and the proximal and distal veins of both lower limbs. Additionally, patients with chest injuries ISS ≥ 2, as well as those with symptoms that raised suspicion for pulmonary embolism, were assessed through CTPA.

In the non-screening group, only patients with clinical symptoms of DVT underwent VUS examination, and patients with clinical features of PE underwent CTPA.

All patients diagnosed with VTE (DVT or PE) received pharmacological treatment of VTE with a therapeutic dose of LMWH. Patients requiring urgent orthopaedic surgery and diagnosed with proximal DVT or PE were referred for the implantation of retrievable inferior vena cava filters (IVCFs). After surgical treatment of the pelvic injury, therapeutic doses of LMWH were continued until the patient attained an ambulatory status. After surgery, both VUS and CTPA were performed in patients with new symptoms, suggesting DVT or PE as appropriate.

Duplex ultrasound was performed using B-mode compression, spectral duplex ultrasound and colour augmentation. All deep and superficial veins of the lower extremities and pelvis were examined. DVT was diagnosed if there was a noncompressible dilated vein or an echogenic thrombus within the vein. Additionally, the absence of or decrease in the blood flow was taken into consideration. VUS was performed by a specialist in radiology within 1–3 days after admission.

The primary outcome measure was in-hospital all-cause mortality. Secondary outcomes included symptomatic and asymptomatic acute PE or DVT.

### 2.4. Data Analysis

The R (4.3.0) statistical package (https://www.r-project.org accessed on 1 April 2023) was used for statistical analyses. Parametric data are reported as the mean ± SD, and nonparametric data are reported as the median (interquartile range). For continuous variables, parametric data were compared using a *t* test, and nonparametric data were compared with the Mann–Whitney U test. Categorical variables were compared using a chi-square or Fisher’s exact test, as appropriate. A *p* value of <0.05 was defined as statistically significant.

## 3. Results

During the study period, 3186 patients with pelvic and/or acetabular fractures were admitted to the Department of Pelvis Injury and Pathologies. There were 897 (28.2%) female and 2289 (71.8%) male patients.

In the first phase (2001–2013), 1975 patients were admitted without screening (control group). There were 548 (27.7%) female and 1427 (72.3%) male patients. The average age was 40 ± 16 years (Table 1).

In the second phase (2013–2021), 1211 patients were admitted to the department and underwent surveillance for VTE (screening group). There were 350 (28.9%) female and 861 (71.1%) male patients. The mean age was 45 (±18) years, which was significantly higher compared to that in the control group (41 ± 16) (*p* < 0.001).

More patients underwent surgical treatment in the non-screening group (75.2% vs. 71%, *p* = 0.008), and individuals in this group were younger (41 vs. 45 years).

The assessment of fracture types for both acetabular (Table 2) and pelvic injuries (Table 3), age and period from injury to surgery across the two groups did not uncover any significant differences. During the study period, surgical approaches evolved. Therefore, there were significant differences between the frequency of surgical approaches used in both groups (Table 4).

There were more VTE cases revealed after admission in the screening group (5.6% vs. 0.9%, *p* < 0.001); in particular, more patients were found to have DVT without PE (3.6% vs. 0.3%, *p* < 0.001) and PE with accompanying DVT (1.6% vs. 0.2%, *p* < 0.001). A higher number of VTE cases resulted in significantly higher numbers of IVCFs placed in patients in the screening group (0.6% vs. 4.5%).

There were no differences between groups in the occurrence of DVT and PE with DVT after surgery. We did not notice any PE without accompanying DVT in the screening group, which was a significant difference compared to the phase without surveillance (0% vs. 0.6%, *p* = 0.022). Nevertheless, the number of patients diagnosed with VTE after surgery was similar in both groups (1.5% vs. 1.5%, *p* = 0.94). The proportion of patients diagnosed with VTE was significantly higher in the surveillance group (6.7% vs. 2%, *p* < 0.001).

There were nine (0.46%) sudden unexplained deaths in the non-screening group. All had symptoms consistent with acute PE. Seven patients died during surgery, and two more fatalities occurred during pre-surgical arrangements. In two (0.1%) cases, a postmortem examination was performed and revealed massive clots in the pulmonary arteries. Since the screening was introduced, we have not encountered any deaths in the perioperative period (0.5 vs. 0, *p* = 0.02).

Seven cases of sudden deaths without postmortem section were analysed. The mean ISS score was 14 ± 10, and the mean age was 59 ± 17. In two cases, there was multi-organ trauma (ISS 34 and 29). In six out of seven cases, upon hospital admission, there were no signs of chronic illnesses detected. In one case, there were comorbidities: hypertension and stable coronary heart disease. All of those had an acetabular fracture, which in one case was associated with a pelvic ring fracture. In light of all factors, we postulate that comorbidities were not essential elements in these fatalities.

DVT was diagnosed in 96 cases during the study period (Table 5). Proximal DVT was found in 17 (17/1975, 0.9%) cases in the non-screening group and in 52 (52/1211, 4.3%) cases in the screening group (*p* > 0.001), while distal DVT was discovered in 4 cases (4/1975, 0.3%) and in 22 cases (22/1211, 1.8%), respectively. Bilateral DVT was detected in seven cases (7/3186, 0.2%), of which, six were distal and one proximal (femoral vv.). VUS revealed three cases of DVT in the limb opposite the fractured acetabulum.

The mean ISS in patients with VTE was 14.3. In the screening group, patients with VTE underwent VUS assessment approximately 19 days after injury and 2.1 days after admission to our hospital.

## 4. Discussion

The results of this study offer a thorough evaluation of VTE events in surgically managed cases of pelvic and acetabular fractures, establishing it as one of the most extensive retrospective analyses available. The application of a screening protocol, which incorporated VUS and CTA in the specific circumstances, yielded a considerably elevated VTE diagnosis rate compared to the group that did not receive screening. The foremost aspect to consider is the substantial decrease in all-cause mortality rate among the monitored group.

Establishing a diagnosis of VTE in patients with pelvic and acetabular fractures depends on several factors, including the diagnostic tool used, such as ultrasound, MRV or CTV, and the frequency of examination (one-time or regular). Furthermore, it is crucial to consider the preventive measures (heparin, LWMH), the timing of the examination (before or after surgery) and the veins that are examined (such as pelvic, proximal and distal veins). Moreover, some authors [16,17,18] suggested that race may also affect the frequency of VTE.

The prevalence of VTE reported in a series without thromboprophylaxis utilising CTA in the Asian population was as high as 27–41% [8,10,19], while the data presented by Fisher et al. [20] suggest a 6–11% rate when employing ultrasound in the United States.

Considering research where VUS was applied and subjects were administered prophylaxis (LWMH), the occurrence of DVT in our results is similar to the results obtained by Hadizie et al. [21] and lower than those published by other authors [9,11,22].

According to Arroyo et al. [23], the Injury Severity Score correlates with the number of VTE cases. In our cohort, the mean ISS in patients with VTE was 14.3, which is much lower than the 27 reported by Steele et al. [22].

The screening protocol showed a higher incidence of distal DVT compared to the non-screening period (33% vs. 17%, *p* < 0.001), similar to other findings [24,25,26]. Results regarding distal DVT reported by Zhao and colleagues were comparable (32%) [9]. Conversely, the research conducted by Olson and colleagues, which included weekly VUS screenings, demonstrated the presence of distal DVT in 65% of high-risk trauma patients [27]. The progression of distal DVT to the proximal segment was found in 3–4.7% of the high-risk trauma patient population [26,28]. Furthermore, Lee and colleagues [29] found that the incidence of PE in patients with proximal and distal DVT is similar (3.3% and 4.7%, respectively). However, the research conducted by Pan and colleagues regarding trauma patients revealed that 17% of cases with pulmonary embolism were identified as the result of distal deep vein thrombosis [30]. The authors also stated that 21.4% of fatal PE cases were caused by distal DVT.

The incidence of bilateral DVT (6/73, 8%) in our study was lower in comparison with the results of Zhao et al. [9] (16%) and Kim et al. [10] (19%). Interestingly in 4% of the cases, DVT was detected in an uninjured limb (3/73), which was also reported by other authors [9].

According to Geerts, compression ultrasonography is the diagnostic test of choice for clinically suspected DVT in patients with pelvic and acetabular fractures [31]. Yet, in select medical contexts, such as those involving extensive lower-extremity wounds or the use of external fixators, the implementation of VUS may be unfeasible, or its outcomes may be deemed inconclusive. Furthermore, the role of this method in visualising pelvic clots remains debatable [32,33]. Alternatively, contrast-enhanced CT or magnetic resonance venography (MRV) may be used. As part of their research, Stannard et al. implemented a screening strategy that involved both VUS and MRV performed within 24 h before discharge in patients with asymptomatic acetabular and pelvic fractures [33]. The researchers found that MRV had a higher success rate in detecting pelvic clots compared to ultrasound, while ultrasound was more accurate in identifying clots in the lower extremities. Abdominal–pelvic CT venography and venous ultrasound were both utilised by Bonhomme et al., and they found that ultrasound as a standalone method may not be enough [11]. The prospective evaluation of CTV and MRV in patients with pelvic and acetabular fractures revealed that diagnostic determinations predicated solely on any of these imaging modalities may lead to unwarranted and forceful interventions as a consequence of the notable rate of erroneous positive outcomes [34]. Nonetheless, a few studies [8,10,19] in patients with pelvic and acetabular fractures have been conducted using CTV for screening the incidence of VTE events.

Venous duplex ultrasound is the preferred means of DVT surveillance, though its incorporation as a screening measure is still a subject of debate [24]. Several studies have suggested that duplex ultrasound (VUS) may not have the requisite accuracy as a screening modality for asymptomatic patients [35,36].

Studies on the utilisation of VUS surveillance as a preventive measure against VTE events in trauma patients have generated divergent outcomes. Research performed by Cipolle and colleagues [37] did not demonstrate a decrease in PE cases among trauma patients through the implementation of regular VUS screening. According to earlier research [38,39,40], the systematic evaluation of at-risk asymptomatic individuals who have experienced trauma may yield therapeutic advantages and is justifiable.

The retrospective analysis of 973 patients with pelvis and acetabulum fractures did not show a difference in the prevalence of PE between the groups screened with VUS and those without screening [41]. Conversely, in light of their research, Steele and colleagues advised the inclusion of preoperative duplex scanning in the treatment protocol for individuals with acetabular fractures who encounter a delay of more than three days before undergoing surgery. They found that this step is crucial for detecting and treating early-onset DVT [22]. It is noteworthy that our investigation revealed that the mean interval between injury and the primary VUS assessment in patients with VTE was 19 days. The primary reason for this delay was concomitant injuries and the time needed for transfer from other hospitals.

According to Moed and colleagues, sequential VUS screening for proximal DVT in asymptomatic patients with pelvic and acetabular fractures did not decrease the risk of PE [42]. The retrospective work of Johnson and colleagues [25] revealed that the probability of PE manifestation is lowered in injured individuals screened with VUS and appropriately treated when VTE was revealed. An observational study by Allen and colleagues [24] on ICU trauma patients with a high risk of VTE (Risk Assessment Profile, RAP ≥ 10) indicated that patients who receive VUS surveillance experience a decrease in PE rates. The findings of Kay and colleagues’ [26] prospective randomised trial, which focused on trauma patients with moderate or high risk (RAP ≥ 5), revealed that the implementation of a selective routine VUS protocol resulted in a notable decrease in in-hospital PE rates. Kay et al. stated that the incorporation of risk stratification is essential in maximising the benefits of regular screening [26]. Nevertheless, Bonhomme et al. found, in pelvic and acetabular patients, no significant difference in the occurrence of a VTE event relative to the preoperative RAP score [11].

Moreover, the primary objective of surveillance VUS is not limited to the prevention of PE and death [24,43]. Regardless of the presence of PE, deep vein thrombosis is a significant complication. As per the findings of Prandoni et al., the collective occurrence of recurrent VTE was 17.5% after 2 years of monitoring [43]. The occurrence of post-thrombotic syndrome can be observed in about 33% of these patients and is strongly connected to recurrent DVTs on the same side. Therefore, it is essential to inform the patient of the need for continued oversight, as post-thrombotic syndrome serves as a further indication for surveillance in this at-risk group [24].

When analysing the results of research conducted in the US, it is important to note that VTE incidence is one of the aspects considered when evaluating treatment quality [26,44,45]. A higher number of DVT and PE diagnoses may lead to a lower hospital rating [26]. Furthermore, the predominant focus of screening for DVT in the US is reducing the occurrence of PE [24,25,26,37,41,46]. Notwithstanding, our method focused on diminishing abrupt mortalities among those in need of surgical procedures.

Our analysis determined that preoperative VUS screening did not lead to a reduction in all PE events. Despite this, when postoperative cases of PE without DVT signs were taken into account, a significant decrease was noted in the screening group. There are several potential explanations for this situation. It has been hypothesised [2,10] that applying pressure to the pelvic veins during surgical procedures may result in the genesis of fresh blood clots, thus posing a risk for the development of both deep vein thrombosis (DVT) and pulmonary embolism (PE). Another interpretation could be that emboli in the pelvic venous system, undetectable by VUS, traversed to the pulmonary arteries. Hence, the implementation of CTA for pelvic veins, as suggested by Bonhomme et al. [11], appears to be a viable resolution for screening for clots. The traditional embolic relationship between DVT and PE has been questioned [13,47,48,49,50]. The theory proposed by Knudson et al. suggests that a blood clot can develop directly within the pulmonary arteries and may not be detectable through a VUS or CTA of the pelvic veins [48].

Nevertheless, none of the mentioned studies [11,24,26] have proven that screening for VTE decreases the sudden death rate in trauma patients. Borer and colleagues’ retrospective analysis of patients with pelvic and acetabular fractures did not show a difference in mortality between the screening and non-screening groups [41]. Furthermore, Bonhomme and colleagues aimed to analyse these patients; however, due to the small number of patients in relation to the incidence of VTE, the main objective could not be achieved [11].

### Limitations

This investigation has several significant limitations. First, the data were collected retrospectively. Hence, this investigation exhibits all the limitations typically seen in a retrospective study of this nature. The study included individuals who received treatment spanning a period of two decades. Over this duration, the heightened awareness of VTE risks among medical professionals, along with advancements in the quality and availability of diagnostic tests, likely contributed to variations in the frequency of VTE diagnoses during the specified study periods. Moreover, most patients were referred to our unit from other health care institutions, causing delays in the screening protocol and, ultimately, in surgical treatment. These parameters limit the applicability of the findings to the entire cohort of trauma patients. Furthermore, data on the initiation and potential delays in the onset of thromboprophylaxis could not be collected. In the non-screening group, ISS was not calculated in all patients; therefore, it could not be compared between groups. Moreover, there were significant differences in the number of patients who underwent surgical treatment. Because of COVID-19, a lower proportion of patients in the screening group may have received surgical treatment. The pandemic led to some patients, especially elderly patients who would otherwise have had surgery, receiving conservative treatment instead [51,52,53]. Nevertheless, we cannot exclude the possibility that patients in the screening group were not as severely traumatised as patients in the non-screening group. There was potential for selection bias in the group of patients being screened. Another difference between the groups was the higher average age in the screened group, which received treatment more recently. This phenomenon was also reported by other investigators [54,55]. The diagnoses in the non-screening group were established through ICD-9 coding and the meticulous evaluation of patient records, though there is potential that some were overlooked. The LMWH utilised for thromboprophylaxis varied in our institution across different periods (enoxaparine, deltaparine or nadroparine).

## 5. Conclusions

Considering these restrictions, our conclusion is that utilising both VUS and CTPA in a standardised protocol is a reliable approach for detecting VTE, leading to reduced mortality rates in individuals with pelvic and acetabular fractures by precisely identifying VTE during the perioperative period.

## Figures and Tables

**Table 1:** Comparison of patients with VTE in the control and the screening groups.

	Non-Screening Group		Screening Group		*p*
No. of patients admitted	1975		1211		
Age, mean ± SD, y	41 (16)		45 (18)		<0.001
Male, n	1427	72.3%	862	71.2%	0.25
DVT after admission, n	5	0.3%	44	3.6%	<0.001
PE with DVT after admission, n	4	0.2%	19	1.6%	<0.001
PE without DVT after admission, n	8	0.4%	5	0.4%	0.973
VTE after admission, n	17	0.9%	68	5.6%	<0.001
Underwent surgery, n	1485	75.2%	859	71%	0.008
DVT after surgery, n	11	0.7%	9	1.1%	0.436
PE with DVT after surgery, n	2	0.1%	4	0.5%	0.126
PE without DVT after surgery, n	9	0.6%	0	0.0%	0.022
VTE after surgery, n	22	1.5%	13	1.5%	0.943
All VTE cases, n	39	2%	81	6.7%	<0.001
IVCF, n	11	0.6%	54	4.5%	<0.001
Mortality, n	9	0.5%	0	0	0.02

SD—standard deviation, y—years, n—number, DVT—deep vein thrombosis, PE—pulmonary embolism, VTE—venous thromboembolism, IVCF—inferior vena cava filter.

**Table 2:** Acetabular fracture types in patients who underwent surgical treatment.

Acetabular Fracture Type	Non-Screening Group		Screening Group		*p*
Period from injury to surgery, mean ± SD, d	19 (21)		18 (13)		0.094
Age, mean ± SD, y	40 (15)		43 (16)		0.103
PW, n	215	20.2%	120	11.3%	0.722
PC, n	43	4%	24	2.3%	0.755
AC, n	19	1.8%	4	0.4%	0.075
AW, n	68	6.4%	40	3.8%	0.677
Tr, n	91	8.5%	48	4.5%	0.927
T, n	68	6.4%	52	4.9%	0.074
PC PW, n	64	6%	36	3.4%	0.878
Tr PW, n	184	17.3%	84	7.9%	0.202
HTr AC, n	37	3.5%	24	2.3%	0.497
BC, n	276	25.9%	144	13.5%	0.767
Total	1065		576		

PW—Posterior Wall, PC—Posterior Column, AC—Anterior Column, AW—Anterior Wall, Tr— Transverse, T—T-type, PC PW—Posterior Column with Posterior Wall, Tr PW—Transverse with Posterior Wall, HTr AC—Associated Anterior Column Posterior Hemitransverse, BC—Associated Both Columns, n—number, d—days, y—years.

**Table 3:** Pelvic fracture type in patients who underwent surgical treatment.

Pelvic Fracture Type	Non-Screening Group		Screening Group		*p*
APC 1, n	9	2.1%	7	2.5%	0.773
APC 2, n	125	29.6%	78	27.6%	0.528
APC 3, n	23	5.5%	11	3.9%	0.928
LC 1, n	26	6.2%	35	12.4%	0.004
LC 2, n	116	27.6%	75	26.5%	0.794
LC 3, n	8	1.9%	4	1.4%	0.773
CMI, n	50	11.9%	32	11.3%	0.813
VS, n	63	15.0%	41	14.5%	0.851
Total	420		283		

APC—Anterior–Posterior Compression, LC—Lateral Compression, CMI—Complex Mechanism of Injury, VS—Vertical Shear.

**Table 4:** Comparison of surgical approaches used in the acetabular fractures in the control and the screening groups.

Acetabular Approach Type	Non-Screening Group		Screening Group		*p*
K-L, n	616	57.8%	262	45.5%	<0.001
I-I, n	218	20.5%	162	28.1%	<0.001
Y, n, n	60	5.6%	1	0.2%	<0.001
I-F + K-L, n	59	5.5%	3	0.5%	<0.001
I-I + K-L, n	66	6.2%	92	16.0%	<0.001
I-F, n	46	4.3%	4	0.7%	<0.001
I-P, n	0	0.0%	52	9.0%	<0.001
Total	1065		576		

K-L—Kocher–Langenbeck, I-I—ilioinguinal, Y—triradiate, I-F— iliofemoral, I-P—intrapelvic.

**Table 5:** Localization of DVT in both groups of patients.

Localisation of DVT		Non-Screening Group	Screening Group	*p*
	Iliac ext. v.	4 (18%)	11 (15%)	0.7
	Femoral v.	10 (45%)	35 (47%)	0.87
	Popliteal v.	3 (14%)	6 (8%)	0.44
Proximal DVT		17 (77%)	52 (70%)	0.52
	Tibial v.	0	1 (1.4%)	0.58
	Peroneal v.	0	11 (15%)	0.06
	Intramuscular vv.	5 (23%)	10 (14%)	0.3
Distal DVT		5 (23%)	22 (30%)	0.52
Total		22	74	

DVT—deep vein thrombosis, ext.—external, v.—vein, vv.—veins.

## Data Availability

Data are contained within the article.

## Abbreviations

VTE	Venous thromboembolism
DVT	Deep vein thrombosis
PE	Pulmonary embolism
VUS	Venous ultrasound
CTPA	Computed tomography pulmonary angiogram
MRV	Magnetic resonance venography
RAP	Risk assessment profile
ISS	Injury severity score
ICU	Intensive care unit
IVCF	Inferior vena cava filter
